# Small-Molecule Tyrosine Kinase Inhibitors Modulate Glucose Handling in C2C12 Cell Line In Vitro: A Mechanistic Study

**DOI:** 10.3390/ph18101445

**Published:** 2025-09-26

**Authors:** Takudzwa Mugiya, Samarah Zvandasara, Mmamosheledi Mothibe, Phikelelani Ngubane, Andile Khathi, Ntethelelo Sibiya

**Affiliations:** 1 Pharmacology Division, Faculty of Pharmacy, Rhodes University, Makhanda 6139, South Africa; takumugiya@gmail.com (T.M.); teamsamarah@gmail.com (S.Z.); m.mothibe@ru.ac.za (M.M.); 2 School of Laboratory Medicine and Medical Sciences, University of KwaZulu-Natal, Durban 4001, South Africa; ngubanep1@ukzn.ac.za (P.N.); khathia@ukzn.ac.za (A.K.)

**Keywords:** diabetes mellitus, small molecule tyrosine kinase inhibitor, glucose uptake, GLUT4 translocation, AKT phosphorylation

## Abstract

**Background:** Small-molecule tyrosine kinase inhibitors (SMTKIs), widely used in cancer chemotherapy, have been reported to variably affect glycaemic control and metabolism, with some agents demonstrating hypoglycaemic effects while others show hyperglycaemic properties. This study aims to elucidate how small-molecule tyrosine kinase inhibitors affect glucose metabolism in C2C12 cells in vitro. Specifically, this study investigated their impact on glucose uptake, AKT expression, GLUT4 expression and translocation, and IL-6 expression. **Methods:** In this study, skeletal muscle (C2C12) preparations were separately treated with small-molecule tyrosine kinase inhibitors; imatinib, dasatinib, axitinib, and erlotinib for 24 h. Thereafter, the effect of the test drugs was assessed on cell viability using the MTT assay, while glucose uptake was determined by measuring residual glucose concentrations in the culture medium with a glucometer. The expression of AKT, GLUT4, and IL-6 and translocation of GLUT4 were evaluated using ELISA. Furthermore, the effect of the drugs was assessed on insulin-stimulated AKT phosphorylation and GLUT4 translocation. Imatinib, dasatinib, axitinib, and erlotinib were selected due to their effect of glucose metabolism, highlighted in the literature. **Results and Discussion:** C2C12 cells treated with SMTKIs were viable after 24 h. A concentration-dependent increase in glucose uptake in C2C12 cells treated with imatinib was observed as the concentration of imatinib increased. Axitinib, dasatinib, and erlotinib demonstrated glucose uptake levels comparable to the control across all concentrations. SMTKIs demonstrated an increase in GLUT4 translocation in the absence of insulin. GLUT4 expression was unchanged in cells treated with small-molecule tyrosine kinase inhibitors compared to the control. Small-molecule tyrosine kinase inhibitors showed an increase in AKT expression. C2C12 cells treated with SMTKI were observed to have elevated IL-6 expression compared to the control. **Conclusions:** The results show that SMTKIs, in particular dasatinib, impact glucose metabolism in C2C12 cells via their effect on GLUT4 translocation and expression and AKT expression. Dasatinib shows promising potential with regard to antidiabetic capabilities. Further research is needed to better understand SMKI effects on metabolic homeostasis, which can perhaps inform future therapeutic strategies.

## 1. Introduction

Diabetes mellitus (DM) is a long-term metabolic ailment associated with insulin deficiency or the inability to respond to insulin effectively. The elevation in DM statistics may be ascribed to sedentary lifestyles and unhealthy diets, especially those rich in refined carbohydrates [1,2]. Reports have estimated that 90% of all DM cases are type 2 DM [3,4]. According to the International Diabetes Federation (IDF), an estimated 537 million adults aged 20–79 years were living with diabetes mellitus globally in 2021. This figure is expected to rise to 643 million (11.3%) by 2030 and to 783 million (12.2%) by 2045 [4]. The DM prevalence has placed an immense burden on health-care systems globally, with 5.2 million death cases globally per year being attributed to this metabolic disorder [5]. Several commonly prescribed therapeutic drugs have been associated with the induction of glucose intolerance or the development of diabetes mellitus (DM) in non-diabetic individuals. In addition, these drugs can disrupt glycaemic control in patients who already have diabetes [6,7]. The impact of these pharmacological modalities on glucose metabolism may be attributed to their capacity to induce insulin resistance, inhibit insulin secretion, and exert direct cytotoxic effects on pancreatic cells [8,9,10]. Scientific reports have demonstrated that patients treated with thiazide diuretics, protease inhibitors, β-blockers, and atypical antipsychotic drugs are at a higher risk of developing DM [11]. Of interest, anti-cancer drugs known as small-molecule tyrosine kinase inhibitors (SMTKIs) have demonstrated varied observations, where some generations have been observed to cause hyperglycaemia, namely nilotinib and ceritinib [12]. On the other hand, other drugs in the same class present hypoglycaemic properties. Studies have shown that SMTIKIs such as imatinib, erlotinib, dasatinib, imatinib, and sunitinib exhibit antihyperglycemic effects, the mechanisms of which are not yet fully understood [13]. Some SMTKIs have been shown to reverse or even prevent type 1 and type 2 DM by reducing insulin resistance and improving β cell dysfunction, thus reversing hyperglycaemia [14]. The literature indicates that SMTKIs can cause hypoglycaemia in both type 1 and type 2 DM [15]. Improvement in HbA1c and glycaemia has been noted in several cases, leading to either termination of or reduction in insulin therapy when patients are being treated with imatinib and sunitinib [16]. A retrospective study investigated the blood glucose concentration in 17 diabetic and 61 non-diabetic patients being treated with imatinib, sunitinib, dasatinib, and sorafenib. A mean decline in blood glucose concentration was observed with the use of all four drugs; imatinib had a mean decline of blood glucose concentration of 9 mg/dL, sunitinib 14 mg/dL, dasatinib 53 mg/dL, and sorafenib 12 mg/dL. The decrease in blood glucose concentration observed in this retrospective study was statistically significant, and it was noted that 47% of patients with diabetes were able to successfully discontinue their diabetic treatment, including insulin therapy [16].

These observations therefore emphasise the necessity for continued research, exploring these anti-cancer agents on their effect on glucose metabolism, offering more holistic insights on their effect on glucose handling and their associated mechanisms of action at both the cellular and molecular levels. We envisage that achieving consensus and a clearer understanding of how these anti-cancer drugs affect glucose handling may provide important insights into whether SMTKIs can be repurposed as antidiabetic agents. With a well-identified mechanism of action and structure–activity relationship, these anti-cancer agents can possibly be repurposed for the management of diabetes. The physicochemical properties, pharmacokinetic, and safety profiles of these agents are already known; therefore, this route could prove to be time-s and cost-effective. Nevertheless, the SMTKI side effect profiles and cost pose significant limitations to their repurposing as chronic antidiabetic therapy. This study investigated the mechanisms of action by which SMTKIs modulate glucose handling. Exploring the effect of these agents, including imatinib, dasatinib, axitinib, and erlotinib, on glucose metabolism in skeletal muscle (C2C12) in vitro could potentially provide a possible mechanistic pathway involved. Our main focus was on the effect of these molecules on glucose uptake and key components of the insulin signalling pathway. Specifically, we examined AKT phosphorylation, GLUT4 expression and translocation, and IL-6 expression using ELISA.

## 2. Results

### 2.1. Cell Viability

**Figure 1:**
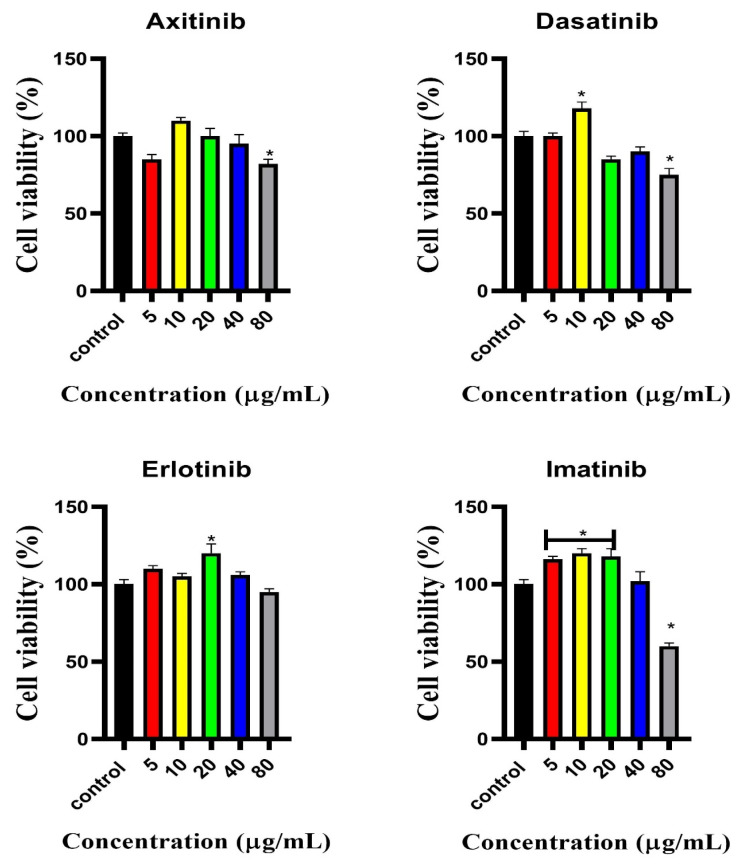
Percentage viability in C2C12 cells treated with SMTKIs (axitinib, dasatinib, erlotinib, and imatinib) at different concentrations 5, 10, 20, 40, and 80 μg/mL. The data is presented as mean ± SD represented with error bars (n = 3), and the asterisk (*) represents the statistical difference between the test compounds and the control at (*p* < 0.05).

A cytotoxicity study was performed in C2C12 cells with SMTKIs (axitinib, dasatinib, erlotinib, and imatinib) at concentrations of 5, 10, 20, 40, and 80 μg/mL, as presented in Figure 1. The control group was assigned a baseline of 100% cell viability. Axitinib exposure, with the exception of the 10 µg/mL concentration, elicited an approximate 10% reduction in cell viability. Statistically significant effects were observed at concentrations of 5 and 80 µg/mL (*p*-values of 0.0399 and 0.0318, respectively). However, cell viability was still maintained above 80%. Cell viability above 80% is considered non-cytotoxic. Dasatinib exposure in C2C12 resulted in a similar trend to axitinib in cell viability, with however, a more pronounced effect on cell viability at 80 µg/mL. Exposure to imatinib and erlotinib demonstrated percentage viability greater than 100% between 5 and 40 μg/mL, imatinib; however, at 80 μg/mL, a percentage viability of 55% was observed with statistical significance. Axitinib, dasatinib, and imatinib demonstrated statistically significant cytotoxic effects at 80 μg/mL.

### 2.2. Estimated Glucose Uptake

**Figure 2:**
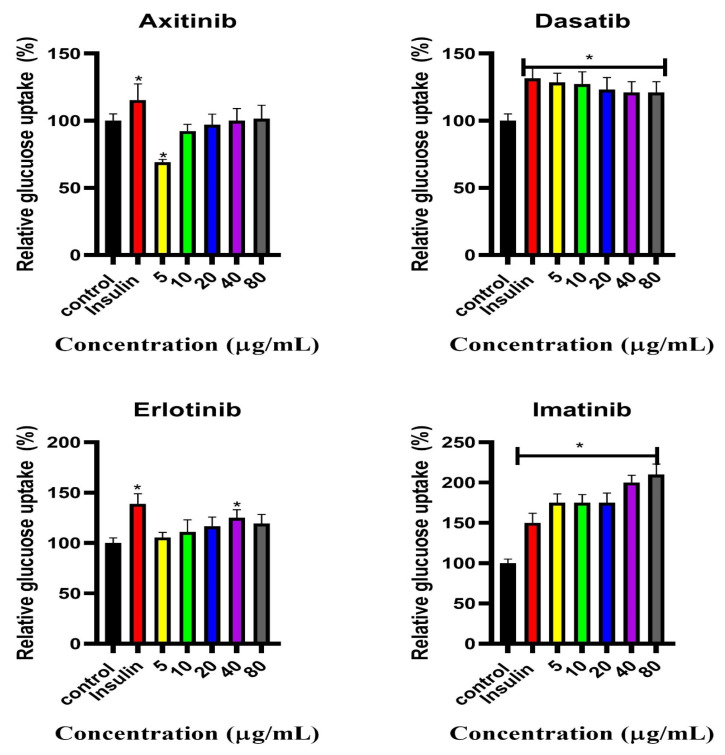
Percentage glucose uptake in C2C12 cells treated with SMTKIs at different concentrations (axitinib, dasatinib, erlotinib, and imatinib): 5, 10, 20, 40, and 80 μg/mL. Control represents sham-treated cells; insulin-treated cells serve as a positive control. The data is presented as mean ± SD represented with error bars (n = 3), and the asterisk (*) represents the statistical difference between the test compounds and the control at (*p* < 0.05).

The percentage glucose uptake was estimated after treating C2C12 cells with SMTKIs (axitinib, dasatinib, erlotinib, and imatinib) at concentrations of 5, 10, 20, 40, and 80 μg/mL (Figure 2). As anticipated, insulin treatment resulted in a significantly elevated percentage of glucose uptake relative to the control condition. Exposure to axitinib resulted in a reduction in glucose uptake by approximately 40% and 10% at concentrations of 5 μg/mL and 10 μg/mL, respectively, compared to the control. At higher concentrations (20–80 μg/mL), glucose uptake levels were comparable to those of the control. In contrast, dasatinib treatment produced a statistically significant increase in glucose uptake at 5 and 10 μg/mL. Erlotinib exposure at concentrations ranging from 5 to 20 μg/mL resulted in glucose uptake levels comparable to the control, with a modest increase of approximately 15%. A statistically significant enhancement in glucose uptake was observed at 40 μg/mL. Imatinib consistently increased glucose uptake across concentrations, with statistically significant elevations compared to the control group.

### 2.3. Effects of SMTKIs (Axitinib, Dasatinib, Erlotinib, and Imatinib) on GLUT4 in C2C12 Cells at Different Concentrations (5, 10, 20, and 80 μg/mL)

#### 2.3.1. GLUT4 Translocation

**Figure 3:**
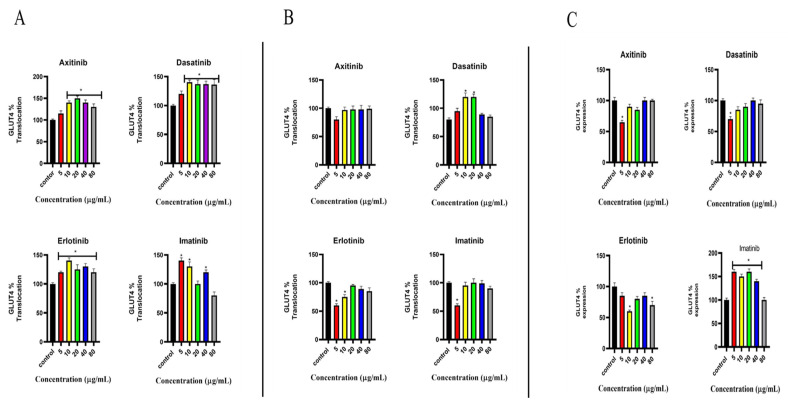
Effects of SMTKIs (axitinib, dasatinib, erlotinib, and imatinib) on GLUT4 in C2C12 cells at different concentrations (5, 10, 20, and 80 μg/mL). Control group represent sham-treated cells and insulin-treated cells. (**A**) Percentage translocation of GLUT4 in C2C12 cells treated with SMTKIs. (**B**) Percentage insulin-stimulated translocation of GLUT4 after SMTKI pre-treatment. (**C**) Percentage expression of GLUT4 in C2C12 cells treated with SMTKIs. The data is presented as mean ± SD with error bars (n = 3). Asterisks (*) indicate statistical differences compared with the control (*p* < 0.05).

Figure 3A illustrates the percentage translocation of the GLUT4 protein in C2C12 cells treated with small-molecule tyrosine kinase inhibitors (SMTKIs). GLUT4 translocation gradually increased in the presence of axitinib, dasatinib, and erlotinib, with statistically significant differences compared to the control as their concentrations increased. In contrast, cells exposed to imatinib exhibited a statistically significant decrease in GLUT4 translocation with increasing concentrations. At the initial concentration (5 μg/mL), imatinib induced a level of GLUT4 translocation that was 40% higher than the control. However, at 80 μg/mL, GLUT4 translocation in imatinib-treated cells was comparable to the control.

#### 2.3.2. Effect of Small-Molecule Tyrosine Kinase Inhibitors on Insulin-Stimulated GLUT4 Translocation

Figure 3B illustrates the percentage of insulin-stimulated GLUT4 translocation in C2C12 cells pre-treated with small-molecule tyrosine kinase inhibitors (SMTKIs). The control group consisted of insulin-treated cells without SMTKI pre-treatment. Compared to the insulin-stimulated control, SMTKI pre-treatment did not significantly affect insulin sensitivity. Dasatinib exposure led to enhanced GLUT4 translocation, with concentrations of 10 and 20 μg/mL inducing an approximate 15% increase relative to control. Consistently, across all SMTKIs investigated, a concentration of 5 µg/mL resulted in a slight reduction in insulin-stimulated GLUT4 translocation.

#### 2.3.3. GLUT4 Expression

Figure 3C depicts the percentage expression of the GLUT4 protein in C2C12 cells treated with small-molecule tyrosine kinase inhibitors. Cells treated with axitinib or dasatinib at a concentration of 5 μg/mL exhibited a statistically significant reduction in GLUT4 expression compared to the control group. However, at higher concentrations ranging from 10 to 80 μg/mL, GLUT4 expression in axitinib-treated cells was comparable to that of the control. In cells treated with erlotinib, a 25% increase in GLUT4 expression was observed as the concentration increased from 5 to 20 μg/mL. In contrast, C2C12 cells exposed to imatinib showed a higher percentage of GLUT4 expression relative to the control group. Notably, as the concentration of imatinib increased from 10 to 40 μg/mL, a corresponding increase in GLUT4 expression was observed.

### 2.4. Effects of SMTKIs (Axitinib, Dasatinib, Erlotinib, and Imatinib) on AKT in C2C12 Cells at Different Concentrations (5, 10, 20, and 80 μg/mL)

#### 2.4.1. AKT Expression

**Figure 4:**
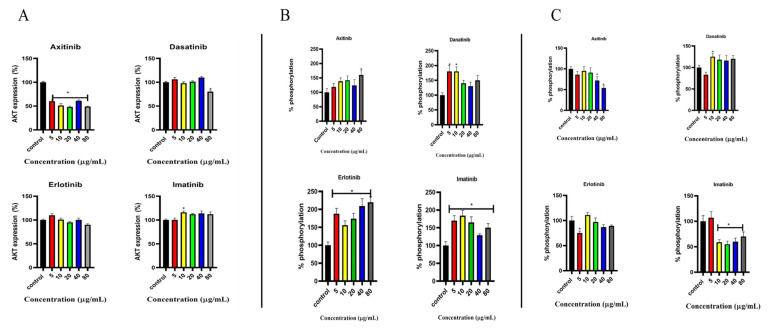
Effects of SMTKIs (axitinib, dasatinib, erlotinib, and imatinib) on AKT in C2C12 cells at different concentrations (5, 10, 20, and 80 μg/mL). Control represents sham-treated cells; insulin-treated cells serve as a positive control. (**A**) Percentage expression of AKT in C2C12 cells treated with SMTKIs. (**B**) Percentage phosphorylation of AKT in C2C12 cells treated with SMTKIs. (**C**) Insulin-stimulated phosphorylation of AKT in C2C12 cells treated with SMTKIs. The data is presented as mean ± SD with error bars (n = 3). Asterisks (*) indicate statistical differences compared with the control (*p* < 0.05).

Figure 4A displays the percentage expression of the AKT protein in C2C12 cells exposed to small-molecule tyrosine kinase inhibitors at concentrations of 5, 10, 20, 40, and 80 μg/mL. In axitinib-treated cells, AKT expression decreased progressively with increasing concentration. Conversely, dasatinib treatment resulted in a concentration-dependent increase in AKT expression. Imatinib exposure led to elevated AKT expression by approximately 8% compared to the control group.

#### 2.4.2. Effect of AKT Phosphorylation

Figure 4B shows the percentage phosphorylation of AKT in C2C12 cells treated with SMTKIs for 24 h. Overall, SMTKI treatment increased AKT phosphorylation compared to untreated controls, with mean increases ranging from 40% to 90%. However, the response did not display a consistent concentration-dependent pattern; for some inhibitors, phosphorylation increased at lower concentrations but plateaued or fluctuated at higher doses, indicating variability across replicates rather than a clear dose–response relationship.

#### 2.4.3. Effect on Insulin-Stimulated AKT Phosphorylation

Figure 4C examines the impact of SMTKIs on insulin-stimulated AKT phosphorylation; the C2C12 were pre-treated with small-molecule kinase inhibitors for 24 h, after which insulin was introduced for one hour followed by analysis of AKT phosphorylation. A control group was not pre-treated with drugs but rather exposed to insulin for one hour. Axitinib (10–80 µg/mL) resulted in a decline in insulin-stimulated AKT phosphorylation. A similar trend was observed for imatinib, at the specified concentrations above. Dasatinib (10–80 µg/mL) pre-treatment led to an increase in insulin-stimulated AKT phosphorylation. Imatinib pretreatment at 10–80 µg/mL had no observable effect on insulin-stimulated AKT phosphorylation. However, a 5% increase in phosphorylation was noted at 5 µg/mL.

### 2.5. Interleukin 6 (IL-6) Cellular Expression

**Figure 5:**
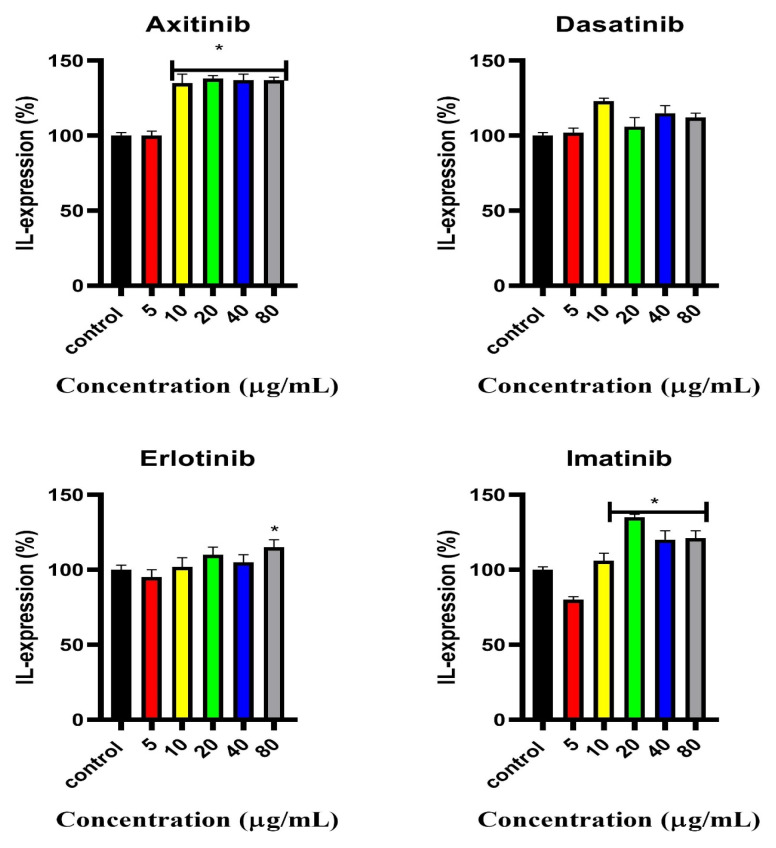
Percentage expression of IL-6 in C2C12 cells treated with SMTKIs (axitinib, dasatinib, erlotinib, and imatinib) at different concentrations 5, 10, 20, 40, and 80 μg/mL. Control represents sham-treated cells; insulin-treated cells serve as a positive control. The data is presented as mean ± SD represented with error bars (n = 3), and the asterisk (*) represents the statistical difference between the test compounds and the control at (*p* < 0.05).

Interleukin-6 (IL-6) expression in C2C12 cells after 24 h treatment with small-molecule tyrosine kinase inhibitors (SMTKIs) was investigated. At lower concentrations (5 μg/mL), IL-6 expression remained comparable to control for most treatments, while higher concentrations (≥10 μg/mL) generally led to significant increases (Figure 5). Erlotinib induced a concentration-dependent increase, reaching significance only at 80 μg/mL, whereas axitinib, dasatinib, and imatinib showed significant effects from mid-to-high concentrations. In particular, axitinib treatment, across all concentrations, resulted in an above 35% increase in IL-6 expression.

### 2.6. Further Studies on Dasatinib

#### 2.6.1. Effect on Wortmannin (PI3K Inhibitor) Exposed Cells

**Figure 6:**
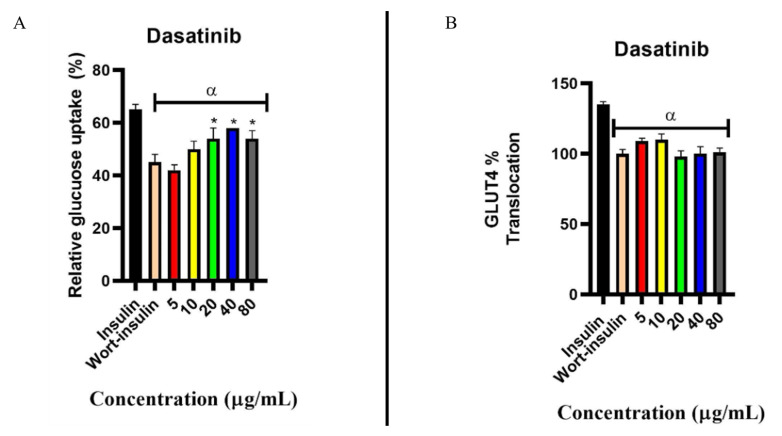
Effects of dasatinib on wortmannin-exposed cells. (**A**) Percentage glucose uptake in C2C12 cells concurrently treated with wortmannin and dasatinib at different concentrations (5, 10, 20, 40, and 80 μg/mL). (**B**) Percentage translocation of GLUT4 in C2C12 cells concurrently treated with wortmannin and dasatinib at different concentrations: 5, 10, 20, 40, and 80 μg/mL. The data is presented as mean ± SD represented with error bars (n = 3), and the asterisk (*) represents the statistical difference between the test compounds and the wort–insulin control at (*p* < 0.05). The alpha (α) represents the statistical difference between the insulin group and other groups.

Figure 6A illustrates the estimated glucose uptake in C2C12 cells following 24 h co-administration of dasatinib and wortmannin. Co-administration of wortmannin and insulin resulted in decreased glucose uptake (20% reduction) compared to cells treated with insulin alone, confirming the inhibitory effect of wortmannin on the insulin signalling pathway. Cells exposed to dasatinib and wortmannin concurrently exhibited reduced glucose uptake relative to the insulin-only group (*p* < 0.05). Interestingly, when compared to the wortmannin–insulin group, dasatinib demonstrated an ability to enhance glucose uptake despite the PI3K inhibitory effect of wortmannin. The concentrations (20–80 µg/mL) were able to increase glucose uptake above 20%, with a significance of *p* < 0.05 The observation may suggest that dasatinib increases glucose uptake utilising an alternative pathway other than the insulin signalling pathway.

Figure 6B further examines the translocation of GLUT4 using the same experimental setup described above. C2C12 cells treated with wortmannin and insulin exhibited a lower percentage (24% reduction) of GLUT4 translocation compared to the insulin-treated group. Dasatinib (5–10 μg/mL) co-administered with wortmannin resulted in a 7% increase in GLUT4 translocation relative to the control, which was not statistically significant. However, as the concentration of dasatinib increased, no further effect was observed, and the observation was comparable to the control.

#### 2.6.2. Effect on Palmitic Acid Exposed Cells

**Figure 7:**
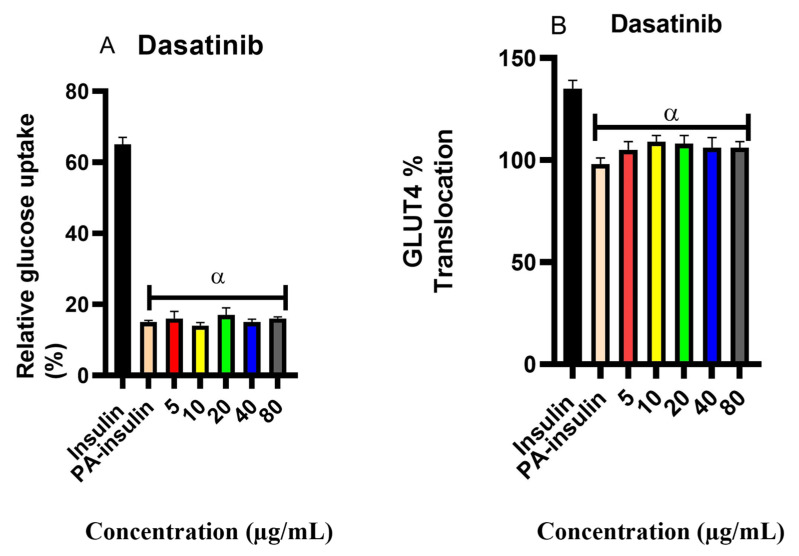
Effects of dasatinib on palmitic acid-exposed cells. (**A**) Percentage glucose uptake in C2C12 cells treated with dasatinib at different concentrations (5, 10, 20, 40, and 80 μg/mL) after exposing cells to palmitic acid. (**B**) Percentage GLUT4 translocation in C2C12 cells treated with dasatinib at different concentrations, 5, 10, 20, 40, and 80 μg/mL, after exposing cells to palmitic acid. The data is presented as mean ± SD represented with error bars (n = 3). The alpha (α) represents the statistical difference between the insulin group and other groups at (*p* < 0.05).

Figure 7A illustrates the effect of dasatinib on cells pre-treated with palmitic acid for 24 h. As expected, insulin treatment of palmitic acid-pretreated cells resulted in a 40% reduction in glucose uptake compared to cells treated with insulin alone (*p* = 1.63 × 10^−6^), confirming a statistically significant impairment of insulin responsiveness. These observations confirmed the insulin resistance state, induced by exposure to palmitic acid. In contrast, dasatinib-treated cells, pre-exposed to palmitic acid, did not significantly alter glucose uptake across all concentrations, as values were comparable to the control group (*p* > 0.05). This, therefore, suggests that dasatinib cannot promote glucose uptake in the palmitic acid-induced insulin-resistant state.

Figure 7B illustrates the percentage translocation of GLUT4 in C2C12 cells following exposure to palmitic acid and subsequent treatment with dasatinib. The experimental setup mirrored that described above. C2C12 cells not exposed to palmitic acid but treated with insulin exhibited a higher percentage of GLUT4 translocation compared to their palmitic acid pre-treated counterparts. These observations further affirm the state of insulin resistance with palmitic acid exposure. Treatment of palmitic acid pre-treated cells with dasatinib showed a modest increase in glucose uptake (<10%), demonstrating no statistical significance.

## 3. Discussion

Literary evidence suggests that a class of anti-cancer drugs known as small-molecule tyrosine kinase inhibitors (SMTKIs) demonstrate mixed alterations of glucose metabolism in patients treated for cancer. Some generations of these agents have been reported to be associated with hypoglycaemia whilst others present with hyperglycaemia. This research study sought to establish the underlying mechanisms through which SMTKIs alter glycaemic control, as reported in the clinical setting. The lowering of blood glucose concentration in patients with cancer being treated with axitinib, dasatinib, erlotinib, and imatinib has been highlighted in numerous studies [17,18]. Interestingly, in some cases, treatment with these specific SMTKIs has led to the discontinuation of antidiabetic medication by patients due to severe hypoglycaemic episodes [19,20]. Given the dysregulation of glucose metabolism observed clinically in patients with cancer, it is prudent that the underlying mechanisms are outlined. In doing so, it could further solidify the repurposing potential of some SMTKIs towards the management of diabetes. In this study, four SMTKIs, namely axitinib, dasatinib, erlotinib, and imatinib, were investigated with the goal of illuminating the mechanisms of action on glucose handling modulation.

SMTKIs inhibit phosphorylation of the receptor tyrosine kinase domain via competitive ATP inhibition. These tyrosine kinase inhibitors disrupt signal transduction pathways of protein kinases. Inhibition of phosphorylation of the receptor at the tyrosine kinase domain by SMTKIs interferes with cell differentiation, proliferation, migration, and survival, and they induce cell apoptosis. SMTKIs are not curative, but they can induce durable remissions by modulating signalling pathways that regulate cellular proliferation and survival. Their effect is on the downstream signalling pathways associated with cell proliferation and differentiation. Hence, we expected the cell viability of the C2C12 cell line not to be drastically affected by exposure to SMTKIs for 24 h. Indeed, our observations suggest that the selected concentrations below 80 µg/mL were tolerated, with the exception of imatinib at the highest concentration. Imatinib has been observed to be cytotoxic in skeletal muscle cells as the concentration increases. This cytotoxicity, in part, stems from mitochondrial dysfunction.

Axitinib is used in the treatment of advanced renal cell carcinoma as a second-line treatment for patients unresponsive to sunitinib [21,22]. In a study conducted on pancreatic adenocarcinoma cells, it was observed that axitinib treatment increased glucose uptake and increased GLUT1 cell surface expression [19]. The effect of axitinib on glucose metabolism remains poorly understood and researched. Likewise, its effects on the insulin signalling pathway are unknown. In our study, axitinib had no effect on glucose uptake. On the contrary, observations from this study suggested that exposure of the C2C12 cell line to axitinib induced the translocation of GLUT4. The GLUT4 translocation observations are in support of the clinical observations, where 5 mg twice daily resulted in decreased blood glucose concentration and HbA1c levels [23]. A possible explanation is that while GLUT4 is translocated, its transporter activity may be functionally impaired, limiting glucose flux despite increased membrane localization. Alternatively, compensatory cellular mechanisms may be engaged, such as downregulation of other glucose transporters. Observations from this study highlight that C2C12 cells treated with axitinib had elevated IL-6 expression. Studies have linked IL-6 to the development of insulin resistance and the pathogenesis of type 2 diabetes mellitus. IL-6 is thought to impair the phosphorylation of the insulin receptor and insulin receptor substrate-1 by promoting the expression of SOCS-3, which is believed to be a potential inhibitor of insulin signalling [24].

Our findings show that SMTKI treatment elevated IL-6 expression in C2C12 cells. The role of IL-6 in glucose metabolism, however, is complex and context-dependent. Chronic IL-6 elevation, such as that observed in obesity or systemic inflammation, has been implicated in the development of insulin resistance [25]. In contrast, transient increases in IL-6, for example, during exercise, have been shown to enhance glucose uptake and improve insulin sensitivity [26]. The IL-6 induction observed in our study may therefore represent an acute cellular stress response, but further work is needed to determine whether this response promotes or impairs insulin signalling in skeletal muscle cells.

For non-small-cell lung cancer, erlotinib is administered at 150 mg once daily, and for pancreatic cancer at 100 mg once daily [27,28]. Case reports have reported an attenuation of hyperglycaemia in patients with non-small-cell lung cancer on erlotinib therapy [20]. This study shows that the C2C12 cell line, when exposed to erlotinib, lead to elevated GLUT4 translocation whilst decreasing overall GLUT4 expression. The observation for GLUT4 did not translate into an increase in glucose uptake, since there was no difference between the treatments and the control. The steady decline in glucose uptake observed in all groups could be attributed to GLUT1, which mediates basal glucose uptake. Skeletal muscle expresses both GLUT4 and GLUT1 transporters. GLUT1, according to Marette et al. (1992), accounts for 5–10% of total glucose carriers in rat skeletal muscle [28]. The dichotomy between GLUT4 membrane translocation and net glucose uptake with erlotinib may suggest that erlotinib reduces GLUT1 abundance/activity or fails to upregulate it [29,30]. Overall uptake of glucose may remain unchanged despite more GLUT4 at the surface.

Imatinib is utilised in the treatment of chronic myeloid leukaemia, gastrointestinal stromal tumour, and other cancers [31,32,33,34]. A case report showed regression of type 2 diabetes mellitus in a patient after long-term imatinib treatment [33]. Gómez-Sámano et al. (2018) observed that patients with gastrointestinal stromal tumour and chronic myeloid leukaemia comorbid with type 2 diabetes mellitus had a considerable reduction in fasting plasma glucose and glycated haemoglobin during imatinib therapy [33]. It has been suggested that imatinib promotes β-cell survival in response to pro-inflammatory cytokines and toxins [35,36]. Based on our study observations, a glucose lowering potential, which could in part explain some clinical observations, was demonstrated. We have demonstrated that imatinib stimulates glucose uptake in skeletal muscle, with both GLUT4 and AKT phosphorylation being central. Interestingly, these observations were attained in the absence of insulin. Typically, the phosphorylation of insulin signalling proteins leads to glucose uptake [32]. An increase in glucose uptake with increasing concentrations of imatinib in the C2C12 cell line was observed. The increase in AKT phosphorylation observed without insulin stimulation points to a possible insulin-independent pathway, which warrants further analysis, perhaps supported by immunofluorescence and sub-cell fractionation studies. The in vitro observations made agree with observations made in numerous retrospective studies where patients on imatinib therapy were noted to demonstrate a reduction in blood glucose concentration, with some patients discontinuing their antidiabetic medication while on imatinib therapy [13,14,15]. Hägerkvist et al. (2006) observed that imatinib had the ability to reduce the effect of several different apoptotic-promoting substances, including the pro-apoptotic MAP kinase JNK, which is linked to inflammation [35]. Interestingly, the IL-6 levels in both cells were observed to have increased as the concentration of imatinib increased. Our IL-6 observations, however, are contrary to those of Huang et al. (2009), who demonstrated a reduction in inflammation by imatinib in mdx mice in vivo, showing a suppression of IL-1β and TNF-α expression [37]. Imatinib increased IL-6 expression while improving glucose uptake in C2C12 cells. This discrepancy could be context-dependent and warrants further investigations. Acutely, an increase in IL-6 has been linked with a reduction in obesity and glucose intolerance [38]. Chronic systemic IL-6 elevation is associated with insulin resistance, while acute IL-6 elevations enhance glucose uptake through AMPK activation [25,26]. Our 24 h, in vitro exposure likely reflects an acute response rather than chronic inflammation; however, this should be a subject of further exploration. Furthermore, in some studies, imatinib demonstrated efficacy in the treatment of immune-related diseases, namely inflammatory bowel disease, autoimmune diabetes, rheumatoid arthritis, and multiple sclerosis [39].

**Figure 8:**
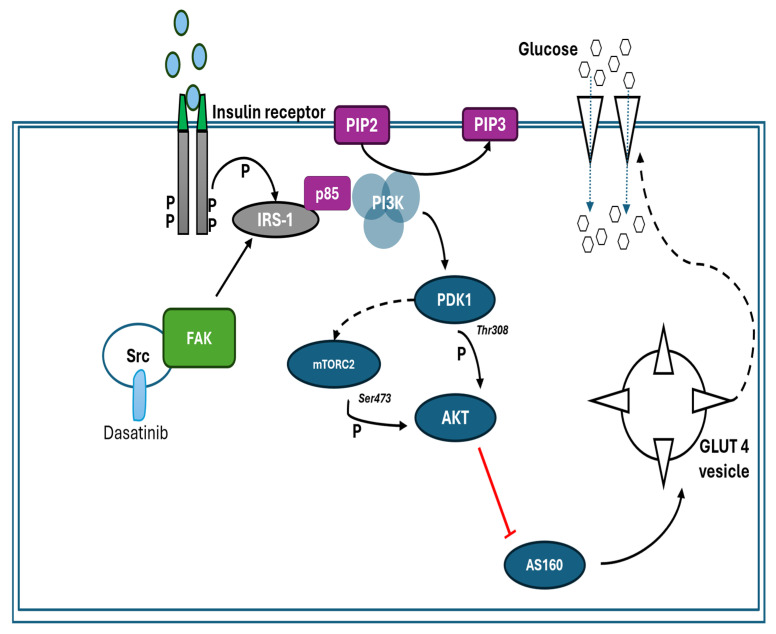
Proposed mechanism of dasatinib. Dasatinib has been demonstrated to increase glucose uptake in skeletal muscle, in part, through an PI3K/AKT-mediated pathway, which results in the translocated of GLUT4. The solid arrows represent the steps in the signalling pathway, which were not investigated in the study. The broken arrows represent processes that are suggested to be affected by SMTKI to enhance glucose uptake in this study.

Dasatinib is an orally administered SMTKI used in the treatment of chronic myeloid leukaemia (CML) and Philadelphia chromosome-positive acute lymphoblastic leukaemia [16,40,41,42]. Concerning the effects of dasatinib on glucose metabolism in patients, there have been mixed observations from studies. Lu Yu et al. (2019), in a retrospective study of 370 patients with chronic myeloid leukaemia, highlighted that the mean fasting glucose level significantly increased in patients who underwent dasatinib therapy for 3 or more months [42]. Some studies have suggested that dasatinib can lower blood glucose concentration in patients undergoing treatment [43,44]. In a case report by Katsumi Iizuka et al., a 63-year-old man on dasatinib treatment showed improvements in the glycaemic index (to less than 6), an improvement in insulin sensitivity, and increased plasma levels of adiponectin and leptin [44]. The clinical antidiabetic properties of dasatinib observed in the studies mentioned above have led to the proposal of dasatinib as a novel diabetes mellitus therapy. Despite these clinical observations, the mechanism(s) through which dasatinib alters glucose metabolism, however, remains unclear. C2C12 cells treatment with dasatinib resulted in enhanced GLUT4 translocation in the C2C12 cell line exposed to insulin and in C2C12 cells where insulin was absent. The enhanced translocation of GLUT4 was more pronounced in the absence of insulin. The elevated GLUT4 translocation in the C2C12 cell line treated with dasatinib, in particular in the absence of insulin, may be an indication that dasatinib might stimulate GLUT4 translocation through an alternative signalling pathway, perhaps the AMPK. An insulin-independent pathway, such as the AMPK pathway, can facilitate GLUT4 translocation [45,46]. AMPK activity was not directly assessed in this study; this interpretation remains speculative and requires further validation. In skeletal muscle, when insulin binds to its cell-surface receptor, it triggers the insulin signalling pathway, leading to the translocation of GLUT4 via activation of the PI3K/AKT pathway [47,48]. GLUT4 is primarily responsible for increased glucose uptake as a response to insulin in peripheral tissues, namely adipose and skeletal muscle [49,50]. The overall effect of dasatinib on the insulin signalling pathway could explain the increase in glucose uptake observed. The overall observations on dasatinib prompted further investigation with the goal to further understand its mechanism of action as far as glucose handling is concerned. For this reason, we further explored the effect of this drug in the presence of wortmannin, a PI3K inhibitor and in a palmitic acid-induced insulin-resistant state. These experiments were aimed at blocking the insulin signalling pathway, which was envisaged to shed more light on its involvement in dasatinib’s glucose-lowering potential. Despite the inhibition of PI3K by wortmannin, dasatinib still showed a modest increase in glucose uptake; however, no effect was observed in GLUT4 translocation, which warrants further investigation. This suggests that glucose uptake, in part, may occur through alternative mechanisms independent of the PI3K/AKT pathway and GLUT4. An example of an alternative mechanism is through GLUT1, which warrants further investigation. However, the inability of dasatinib to stimulate GLUT4 translocation in the presence of PI3K inhibitor may suggest that this drug, in part, employs the PI3K/AKT pathway. Interestingly, dasatinib had no effect on glucose uptake and GLUT4 in palmitic acid-induced insulin-resistant cells. The failure of dasatinib in palmitic acid-induced insulin-resistant cells could perhaps allude to the lack of functional PI3/AKT pathway in this model, as this is a well-established in vitro insulin resistance model. Taken together, the observation from both wortmannin- and palmitic acid-exposed cells suggest that dasatinib may be utilising the PI3K/AKT pathway to mediate the glucose lowering effect (Figure 8). However, further studies are warranted to ascertain the involvement of GLUT1, MAPK, and AMPK pathways, amongst others. Furthermore, more studies on other insulin in vitro insulin resistance models, including chronic high-insulin- or TNF-a-exposed cell models, are necessary to provide consolidated understanding. Future studies could also benefit from using robust imaging techniques especially for expression and localization studies. Moreover, our study relied on an indirect measurement of glucose uptake, which could be strengthened by directly fluorescence-based glucose uptake techniques in the future.

Diabetes prevalence still demonstrates an upward trajectory; therefore, potential antidiabetic drugs, especially those whose safety and pharmacokinetic profiles have been recognised, should be leveraged. Findings from this study and other previous studies highlight that SMTKIs could be repurposed towards diabetes mellitus treatment. This is supported by their demonstrated ability to enhance glucose uptake and AKT activation in skeletal muscle cells independent of insulin. Potential mechanisms may involve interactions between insulin signalling and alternative pathways such as AMPK activation, GLUT1-mediated glucose transport, or modulation of inflammatory cytokines like IL-6. However, these mechanistic insights are derived from acute in vitro observations, and their translation to clinical outcomes remains uncertain. Long-term in vivo studies will be useful in determining whether these effects are sustained, whether they translate into meaningful improvements in glycaemic control, and how they balance against the adverse effects associated with chronic use of SMTKIs. In summary, our findings highlight that SMTKIs differentially modulate glucose uptake and signalling in skeletal muscle cells. Dasatinib exhibited insulin-independent effects. These results underscore the complexity of kinase inhibitor actions on metabolic processes, with implications for drug-induced dysglycemia in clinical settings. Future studies should focus on identifying the specific glucose transporters and signalling intermediates responsible for PI3K-independent uptake, as well as validating AMPK’s role in dasatinib-mediated GLUT4 regulation. Elucidating these mechanisms will provide deeper insights into the metabolic consequences of targeted cancer therapies and may inform strategies to mitigate adverse metabolic effects. Moreover, the observations from this work further underscore the necessity to closely monitor glycaemic aberrations in cancer patients on SMTKIs. Whilst repurposing dasatinib could be a promising avenue towards diabetes management, the side effect effects presented by dasatinib should not be overlooked. Dasatinib may present with pulmonary complications such as pulmonary hypertension and pleural effusion, amongst others [51]. Considering that diabetes is a chronic disease, these undesirable effects could negate repurposing calls. Lastly, other reports have indicated hyperglycaemia with the use of dasatinib, which calls for further exploration, aiming to corroborate epidemiological, clinical, and experimental data to achieve the consensus. Nevertheless, understanding how these drugs cause antihyperglycemic effects could provide further insight into their pharmacological targets as far as glucose handling is concerned. Furthermore, through these insights, SMTKIs may provide pharmacophores to develop novel agents towards the management of DM.

## 4. Materials and Methods

### 4.1. Drugs and Chemicals

All drugs and chemicals used in the study were of pharmaceutical and chemical grade. The following drugs and reagents were sourced at Merck, Johannesburg, South Africa, a division of Sigma. The following chemicals and drugs were purchased from Sigma-Aldrich, St Louis, MO, USA: Dulbecco’s modified eagle’s medium (DMEM) (D0822), foetal bovine serum (FBS) (12007C), penicillin–streptomycin (P4458), rabbit polyclonal anti-phospho-AKT antibody (SAB4503853), anti-AKT2 (SAB4500798), GLUT4 antibody (ZRB1240), Anti-IL-6 (ZRB1970). tetramethylbenzidine (TMB) (T0440), horseradish peroxidase (HRP) secondary antibody (R5506), and 3-(4,5-dimethylthiazol-2-yl)-2,5-diphenyl-2H-tetrazolium bromide (MTT) (475989).

- Axitinib (CDS023389): *N*-methyl-2-[3-((*E*)-2-pyridin-2-yl-vinyl)-1*H*-indazol-6-ylsulfanyl]-benzamide.
- Dasatinib (SML2589): *N*-(2-chloro-6-methylphenyl)-2-[[6-[4-(2-hydroxyethyl)-1-piperazinyl]-2-methyl-4-pyrimidinyl]amino]-1,3-thiazole-5-carboxamide.
- Erlotinib (SML2156): *N*-(3-ethynylphenyl)-6,7-bis(2-methoxyethoxy)quinazolin-4-amine.
- Imatinib (SML1027). *N*-(4-methyl-3-((4-(pyridin-3-yl)pyrimidin-2-yl)amino)phenyl)-4-((4-methylpiperazin-1-yl)methyl)benzamide.

The purities of the SMTKIs were greater than 98% (HPLC).

### 4.2. Preparation of Test Drugs

The stock solutions of erlotinib, imatinib, dasatinib, and axitinib were prepared by solubilising in DMSO (0.1%) and made up to 1 mL by Dulbecco’s modified eagle’s medium (DMEM). Before each assay, stock solutions were prepared to the desired concentrations (5, 10, 20, 40, and 80 μg/mL) using DMEM.

### 4.3. Cell Culture

The assays were conducted using C2C12 skeletal muscle cells. The C2C12 cells were cultured in a humidified incubator with 5% CO_2_ at 37 °C, in tissue culture flasks (T25 and T75). The C2C12 cells were maintained in DMEM supplemented with FBS (10%) and penicillin–streptomycin (1%). After the cells had grown to confluence (approximately 80%), they were trypsinized and then transferred into new flasks until ready for seeding into either 24 or 96 plates.

### 4.4. Skeletal Muscle Differentiation

After seeding, the skeletal muscle cells (C2C12) were further differentiated to form myotubes. C2C12 myoblast differentiation into myotubes was attained by switching the medium from DMEM supplemented with FBS (10%) to DMEM supplemented with FBS (0.2%) and penicillin–streptomycin (1%) for four days. The media was changed daily, and the extent of differentiation was observed and confirmed under an inverted microscope. The morphological features used to confirm differentiation were striation patterns, elongation, and cell fusion.

### 4.5. Cell Viability

To assess cell viability, the 3-(4,5-dimethyl-2-thiazolyl)-2,5-diphenyl-2*H*-tetrazolium bromide (MTT) assay was conducted in the presence of selected drugs (axitinib, dasatinib, erlotinib, and imatinib). C2C12 cells were seeded into 96-well clear-bottom tissue culture plates at a density of 4.65 × 10^4^ cells/mL and cultured until they reached approximately 80% confluence. The cell preparations were separately exposed to different concentrations (5, 10, 20, 40, and 80 μg/mL) of the SMTKIs. After 24 h, an MTT solution (200 µL, 5 mg/mL), prepared by dissolving in PBS (10%) and FBS-free media (90%), was added into each well. The plate was then incubated for three hours in the dark at 37 °C. Thereafter, the media was removed and replaced with DMSO (200 μL), followed by incubation for five minutes. The absorbance was measured at 570 nm using a UV-VIS spectrophotometer [12]. The assay was performed in triplicates and repeated twice. The cell viability was calculated as follows:

$$Percentage\;viability\;(\%)\;=\frac{absorbance\;of\;sample}{absorbance\;of\;control}\;\times \;100$$

### 4.6. Accu-Check Performa Glucometer Appraisal and Validation for Glucose Uptake Measurement

Bayer Accu-Check Performa glucometers and the associated test strips are commercially utilised to measure blood glucose concentrations. To validate the use of these glucometers and glucose stripes for measuring cell culture medium glucose, the following validation procedure was performed. The glucose concentration of the cell culture medium was initially calculated based on its composition. The values were then converted to mmol/L, the unit used by the glucometer for glucose measurements. Thereafter, 4 mL of cell culture medium was aspirated from the stock solution and then serially diluted to make the following theoretical concentrations: 24.97, 12.48, 6.24, 3.12, and 1.56 mmol/L. Then the glucometer was used to record the actual glucose concentration of the dilutions. The results, shown in the Supplementary Material (Supplementary Figure S1), show glucometer validation for the media glucose concentration measurement experiment: theoretical glucose concentration vs. glucometer glucose reading. The R^2^ value observed from the plot is 0.9972. The R^2^ is very close to 1, indicating that the regression line approximates the actual data very well. This means that the glucometer readings are statistically similar to the theoretical glucose concentrations, hence the glucometer was used to estimate cellular glucose uptake. These findings validate the use of glucometers and test strips for relative comparisons of glucose concentrations.

### 4.7. Estimation of Cellular Glucose Uptake

The glucose uptake was estimated by measuring the concentration of glucose present in the medium after 24 h, according to the method described by Cruz-Bermúdez et al. [52]. To achieve this, C2C12 (3.96 × 10^4^) cells/mL were plated in 24-well plates and left to adhere and reach confluence. The C2C12 cells were differentiated as described above. Thereafter, the culture medium was aspirated from the wells and washed with phosphate-buffered saline (PBS) (200 μL) three times. Medium glucose concentration was measured before incubation with drugs, to serve as Time 0 (T0), 24 mmol/L. Thereafter, the C2C12 cells were treated with the selected drugs at 5, 10, 20, 40, and 80 μg/mL in a medium supplemented with 10% FBS and 1% penicillin–streptomycin and preincubated at 37 °C for 24 h (T24). The cells receiving only a medium concentration (200 µL) served as the control, while insulin (0.05 units) was utilised as a standard drug. After the 24 h incubation period, the medium glucose concentration was measured using an Accu-Check Performa glucometer (Roche, Midrand, South Africa) [13]. The assay was performed in triplicates and repeated twice. The estimation of glucose uptake was determined using the following formula:

$$Glucose\;uptake\;(\%)\;=\frac{Medium\;glucose\;\left(T0\right)-Medium\;glucose\;(T24)}{Medium\;(T0)}\;\times \;100$$

### 4.8. In-Cell ELISA

C2C12 (4.09 × 10^4^) cells/mL were seeded into 96-well plates until they reach appropriate confluency and differentiation. The cells were treated as indicated above. In-cell ELISA assays were conducted to investigate the expression of AKT, GLUT4, and IL-6, as well as the translocation of GLUT4 and AKT phosphorylation. After treatments, the medium was aspirated, and paraformaldehyde (100 μL of 8%) was added to each well for cell fixation. The plate was then incubated for 15 min at room temperature on a microplate shaker set at 300 rpm. Afterwards, the paraformaldehyde was aspirated, and each well was washed four times with PBS (200 μL). In total, 200 μL of 2× permeabilization buffer containing Triton X-100 (250 μL in 24.75 mL 1X PBS) was added to each well, followed by incubation for 30 min at room temperature with shaking at 300 rpm. Thereafter, the blocking process was conducted by the addition of 200 μL of blocking buffer (1X BSA dissolved in PBS), which was added to each well and left to incubate at room temperature for 2 h while shaking (300 rpm). Afterwards, the blocking buffer was aspirated, and 100 μL of primary antibody (anti-GLUT4, anti-AKT, or anti-IL-6) in an incubation buffer was added to each well separately. The plate was then incubated overnight at 4 °C. Next, 100 μL of a secondary antibody (anti-rabbit IgG) specific to the primary antibody was added to each well and incubated for 2 h. Thereafter, 100 μL of horseradish peroxidase substrate (HRP) (0.05 mg/mL) was transferred into each well and incubated for 30 min. To stop the reaction, HCL (100 μL, 0.1 M) was added into each well, and absorbance was read at 450 nm using a spectrophotometer. The assays were performed in triplicates. To determine GLUT4 translocation, the same procedure was followed but without the cell permeabilization step in order to capture only GLUT4 on the cell surface. The relative percentage expression, phosphorylation, or translocation was calculated as follows:

$$Relative\;percentage\;(\%)=\frac{Average\;absorbance\;of\;treated\;wells}{Average\;absorbance\;of\;control}\;\times \;100$$

### 4.9. Insulin-Stimulated AKT Phosphorylation and GLUT4 Translocation

SMTKIs were assessed on insulin sensitivity. To achieve this, the differentiated C2C12 were pre-treated with the selected drugs for 24 h. Thereafter, the media containing drugs was discarded, and the cells were washed with pre-warmed PBS, before FBS-free media containing insulin was introduced into the cells and incubated for one hour. Thereafter, the cell preparations were fixed using paraformaldehyde, followed by an in-cell ELISA aiming to capture insulin-stimulated phosphorylated AKT and GLUT4 translocation. The relative percentage of either insulin-stimulated AKT phosphorylation or GLUT4 translocation was calculated using the above equation.

### 4.10. Further Studies on Dasatinib

Based on initial findings on the overall activity, dasatinib was selected to further understand whether it may be utilising alternative pathways other than the insulin signalling pathway. To achieve this, dasatinib was investigated in the presence of a PI3K inhibitor (Wortmannin), and in palmitic acid-pretreated cells. The premise for these studies was investigating the effect of dasatinib in conditions where the insulin signalling pathway has been inhibited. This investigation aimed at demonstrating whether dasatinib could be employing the insulin signalling pathway or an alternative pathway.

#### 4.10.1. Wortmannin Exposed Cells

The C2C12 cells were plated and differentiated in 24-well plates, where glucose concentration measurements were performed following co-administration of dasatinib (5, 10, 20, 40, and 80 μg/mL) and wortmannin 25 nm for 24 h. Control groups consisted of cells treated wortmannin and insulin and cells treated only with insulin. Glucose uptake estimation and GLUT4 translocation were performed as described above. For GLUT4 translocation studies, the in cell-ELISA described above was conducted.

#### 4.10.2. Palmitic Induced Insulin Resistance Cells

Differentiated C2C12 cells plated in 24-well plates were exposed to palmitic acid (250 μM) for 24 h to induce insulin resistance, as previously described [53]. Control groups consisted of cells not exposed to palmitic acid but treated with insulin and cell exposed to palmitic acid and treated with insulin. Following palmitic acid-induced insulin resistance, the cells were treated with dasatinib at 5, 10, 20, 40, and 80 μg/mL. Glucose uptake estimation and GLUT4 translocation were performed as described above.

### 4.11. Statistical Analysis

Each experiment was performed in triplicate and repeated twice to ensure reproducibility, and the data is presented as means ± SDs on separate column graphs for clarity and represented by an error bar (n = 3). For each experimental condition, biological replicates were defined as independent experiments performed on different passages of C2C12, and technical replicates referred to parallel wells under identical conditions. Standard assumptions were tested before ANOVA. Statistical analysis was performed using GraphPad prism, version 10.6.0. One-way analysis of variance (ANOVA) trailed by Tukey post-test was used to test for statistical significance difference at *p*-values ≤ 0.05. The results of these analyses are presented on the corresponding graphs, with asterisks (*) denoting statistically significant differences (*p* ≤ 0.05) between the control and treatment groups.

## 5. Conclusions

The development of SMTKIs has transformed cancer therapy, and growing evidence indicates that they may also influence glucose metabolism. In this study, imatinib, dasatinib, axitinib, and erlotinib were assessed for their effects on skeletal muscle glucose handling. Our findings demonstrate that certain SMTKIs, particularly imatinib and dasatinib, enhanced glucose uptake, in part through increased GLUT4 translocation and AKT phosphorylation, key components of the insulin signalling pathway. These mechanistic insights provide a clearer understanding of how SMTKIs may improve glycaemic control.

Importantly, our results align with case reports and retrospective studies that describe improved glycaemic profiles and even regression of type 2 diabetes in patients receiving imatinib therapy. While these data highlight the translational potential of repurposing selected SMTKIs for diabetes management, validation in insulin-resistant animal models, human primary skeletal muscle cells, and ultimately clinical trials remains essential.

Taken together, this study not only identifies specific SMTKIs with antidiabetic potential under defined in vitro conditions but also establishes a foundation for future mechanistic and translational research aimed at broadening their therapeutic relevance beyond oncology.

## Data Availability

The original contributions presented in this study are included in the article. Further inquiries can be directed to the corresponding author.

## Supplementary Materials

The following supporting information can be downloaded at: https://www.mdpi.com/article/10.3390/ph18101445/s1, Figure S1: Glucometer validation for media glucose concentration measurement Experiment; theoretical glucose concentration vs. glucometer glucose reading.

## Abbreviations

The following abbreviations are used in this manuscript:

SMTKI	Small-molecule tyrosine kinase inhibitor
GLUT4	Glucose transporter 4
AKT	Ak murine thymoma

## References

[1] Bastaki S.S. (2005). Diabetes mellitus and its treatment. Int. J. Diabetes Metab..

[2] Saeedi P., Petersohn I., Salpea P., Malanda B., Karuranga S., Unwin N., Colagiuri S., Guariguata L., Motala A.A., Ogurtsova K. (2019). Global and regional diabetes prevalence estimates for 2019 and projections for 2030 and 2045: Results from the International Diabetes Federation Diabetes Atlas, 9th edition. Diabetes Res. Clin. Pract..

[3] Sibiya N., Mzimela N., Mbatha B., Ngubane P., Khathi A. (2022). The Insights on Why Diabetes Prevalence May Increase Amid or Post COVID 19 Pandemic. Curr. Diabetes Rev..

[4] Magliano D.J., Boyko E.J., IDF Diabetes Atlas 10th Edition Scientific Committee (2021). Chapter 3, Global picture. IDF Diabetes Atlas [Internet].

[5] Glovaci D., Fan W., Wong N.D. (2019). Epidemiology of Diabetes Mellitus and Cardiovascular Disease. Curr. Cardiol. Rep..

[6] Fève B., Scheen A.J. (2022). When therapeutic drugs lead to diabetes. Diabetologia.

[7] Bressler P., DeFronzo R.A. (1994). Drugs and diabetes. Diabetes Rev..

[8] Fathallah N., Slim R., Larif S., Hmouda H., Ben Salem C. (2015). Drug-Induced Hyperglycaemia and Diabetes. Drug Saf..

[9] Alwhaibi M., Balkhi B., Alhawassi T.M., Alkofide H., Alduhaim N., Alabdulali R., Drweesh H., Sambamoorthi U. (2018). Polypharmacy among patients with diabetes: A cross-sectional retrospective study in a tertiary hospital in Saudi Arabia. BMJ Open.

[10] Dobrică E.-C., Găman M.-A., Cozma M.-A., Bratu O.G., Pantea Stoian A., Diaconu C.C. (2019). Polypharmacy in Type 2 Diabetes Mellitus: Insights from an Internal Medicine Department. Medicina.

[11] Izzedine H., Launay-Vacher V., Deybach C., Bourry E., Barrou B., Deray G. (2005). Drug-induced diabetes mellitus. Expert Opin. Drug Saf..

[12] Oh J.J., Hong S.K., Joo Y.M., Lee B.K., Min S.H., Byun S.-S., Lee S.E. (2012). Impact of Sunitinib Treatment on Blood Glucose Levels in Patients with Metastatic Renal Cell Carcinoma. jpn J. Clin. Oncol..

[13] Fountas A., Diamantopoulos L.N., Tsatsoulis A. (2015). Tyrosine Kinase Inhibitors and Diabetes: A Novel Treatment Paradigm?. Trends Endocrinol. Metab..

[14] Hadova K., Mesarosova L., Kralova E., Doka G., Krenek P., Klimas J. (2021). The tyrosine kinase inhibitor crizotinib influences blood glucose and mRNA expression of GLUT4 and PPARs in the heart of rats with experimental diabetes. Can. J. Physiol. Pharmacol..

[15] Buffier P., Bouillet B., Smati S., Archambeaud F., Cariou B., Verges B. (2018). Expert opinion on the metabolic complications of new anticancer therapies: Tyrosine kinase inhibitors. Ann. d’Endocrinologie.

[16] Agostino N.M., Chinchilli V.M., Lynch C.J., Koszyk-Szewczyk A., Gingrich R., Sivik J., Drabick J.J. (2011). Effect of the tyrosine kinase inhibitors (sunitinib, sorafenib, dasatinib, and imatinib) on blood glucose levels in diabetic and nondiabetic patients in general clinical practice. J. Oncol. Pharm. Pract..

[17] Althubiti M. (2022). Tyrosine Kinase Targeting: A Potential Therapeutic Strategy for Diabetes. Saudi J. Med. Med. Sci..

[18] Duggan B.M., Marko D.M., Muzaffar R., Chan D.Y., Schertzer J.D. (2023). Kinase inhibitors for cancer alter metabolism, blood glucose, and insulin. J. Endocrinol..

[19] Hudson C.D., Hagemann T., Mather S.J., Avril N. (2014). Resistance to the tyrosine kinase inhibitor axitinib is associated with increased glucose metabolism in pancreatic adenocarcinoma. Cell Death Dis..

[20] Onder A.H., Heybeli C. (2024). Improvement of Hyperglycemia Following Treatment with Erlotinib. Endocrine.

[21] Escudier B., Gore M. (2011). Axitinib for the management of metastatic renal cell carcinoma. Drugs RD.

[22] Chen Y., Tortorici M.A., Garrett M., Hee B., Klamerus K.J., Pithavala Y.K. (2013). Clinical Pharmacology of Axitinib. Clin. Pharmacokinet..

[23] Tyrrell H.E.J., Pwint T. (2014). Sunitinib and improved diabetes control. BMJ Case Rep..

[24] Benrick A., Wallenius V., Asterholm I.W. (2012). Interleukin-6 mediates exercise-induced increase in insulin sensitivity in mice. Exp. Physiol..

[25] Rehman K., Akash M.S.H., Liaqat A., Kamal S., Qadir M.I., Rasul A. (2017). Role of Interleukin-6 in Development of Insulin Resistance and Type 2 Diabetes Mellitus. Crit. Rev. Eukaryot. Gene Expr..

[26] Tang P.A., Tsao M.S., Moore M.J. (2006). A review of erlotinib and its clinical use. Expert Opin. Pharmacother..

[27] Lampson B.L., Nishino M., Dahlberg S.E., Paul D., Santos A.A., Jänne P.A., Oxnard G.R. (2016). Activity of erlotinib when dosed below the maximum tolerated dose for EGFR-mutant lung cancer: Implications for targeted therapy development. Cancer.

[28] Marette A., Richardson J.M., Ramlal T., Balon T.W., Vranic M., Pessin J.E., Klip A. (1992). Abundance, localization, and insulin-induced translocation of glucose transporters in red and white muscle. Am. J. Physiol..

[29] Zorzano A., Muñoz P., Camps M., Mora C., Testar X., Palacín M. (1996). Insulin-induced redistribution of GLUT4 glucose carriers in the muscle fiber: In search of GLUT4 trafficking pathways. Diabetes.

[30] Lyseng-Williamson K., Jarvis B. (2001). Imatinib. Drugs.

[31] Peng B., Lloyd P., Schran H. (2005). Clinical Pharmacokinetics of Imatinib. Clin. Pharmacokinet..

[32] Veneri D., Franchini M., Bonora E. (2005). Imatinib and regression of type 2 diabetes. N. Engl. J. Med..

[33] Gómez-Sámano M.Á., Baquerizo-Burgos J.E., Coronel M.F.C., Wong-Campoverde B.D., Villanueva-Martinez F., Molina-Botello D., Avila-Rojo J.A., Palacios-Báez L., Cuevas-Ramos D., Gomez-Perez F.J. (2018). Effect of imatinib on plasma glucose concentration in subjects with chronic myeloid leukemia and gastrointestinal stromal tumor. BMC Endocr. Disord..

[34] Sanz-González S.M., Castro C., Pérez P., Andrés V. (2004). Role of E2F and ERK1/2 in STI571-mediated smooth muscle cell growth arrest and cyclin A transcriptional repression. Biochem. Biophys. Res. Commun..

[35] Hägerkvist R., Makeeva N., Elliman S., Welsh N. (2006). Imatinib mesylate (Gleevec) protects against streptozotocin-induced diabetes and islet cell death in vitro. Cell Biol. Int..

[36] Timper K., Denson J.L., Steculorum S.M., Heilinger C., Engström-Ruud L., Wunderlich C.M., Rose-John S., Wunderlich F.T., Brüning J.C. (2017). IL-6 Improves Energy and Glucose Homeostasis in Obesity via Enhanced Central IL-6 trans-Signaling. Cell Rep..

[37] Huang P., Zhao X.S., Fields M., Ransohoff R.M., Zhou L. (2009). Imatinib attenuates skeletal muscle dystrophy in mdx mice. FASEB J..

[38] Kim H., Kim C.Y., Kim D., Kim E., Ma L., Park K., Liu Z., Huang K., Wen W., Ko J. (2024). Protective Effects of Imatinib on a DSS-induced Colitis Model Through Regulation of Apoptosis and Inflammation. Vivo.

[39] Azizi G., Mirshafiey A. (2013). Imatinib mesylate: An innovation in treatment of autoimmune diseases. Recent Pat. Inflamm. Allergy Drug Discov..

[40] Lindauer M., Hochhaus A. (2018). Dasatinib. Small Molecules in Hematology.

[41] Araujo J., Logothetis C. (2010). Dasatinib: A potent SRC inhibitor in clinical development for the treatment of solid tumors. Cancer Treat. Rev..

[42] Yu L., Liu J., Huang X., Jiang Q. (2019). Adverse effects of dasatinib on glucose-lipid metabolism in patients with chronic myeloid leukaemia in the chronic phase. Sci. Rep..

[43] Salaami O., Kuo C.-L., Drake M.T., Kuchel G.A., Kirkland J.L., Pignolo R.J. (2021). Antidiabetic Effects of the Senolytic Agent Dasatinib. Mayo Clin. Proc..

[44] Iizuka K., Niwa H., Kato T., Takeda J. (2016). Dasatinib improves insulin sensitivity and affects lipid metabolism in a patient with chronic myeloid leukaemia. Case Rep..

[45] Long Y.C., Zierath J.R. (2006). AMP-activated protein kinase signaling in metabolic regulation. J. Clin. Investig..

[46] Watson R.T., Pessin J.E. (2006). Bridging the GAP between insulin signaling and GLUT4 translocation. Trends Biochem. Sci..

[47] Galante P., Mosthaf L., Kellerer M., Berti L., Tippmer S., Bossenmaier B., Fujiwara T., Okuno A., Horikoshi H., Häring H.U. (1995). Acute hyperglycemia provides an insulin-independent inducer for GLUT4 translocation in C2C12 myotubes and rat skeletal muscle. Diabetes.

[48] He A., Liu X., Liu L., Chang Y., Fang F. (2007). How many signals impinge on GLUT4 activation by insulin?. Cell. Signal..

[49] Koester A.M., Geiser A., Bowman P.R.T., van de Linde S., Gadegaard N., Bryant N.J., Gould G.W. (2022). GLUT4 translocation and dispersal operate in multiple cell types and are negatively correlated with cell size in adipocytes. Sci. Rep..

[50] Faria B.Q., Calixto P.S., Picheth G., Ferreira L.M., Rego F.G.d.M., Guerra J.F.d.C., Sari M.H.M. (2025). Palmitate-induced hepatic insulin resistance as an in vitro model for natural and synthetic drug screening: A scoping review of therapeutic candidates and mechanisms. Chem. Biol. Interact..

[51] Nekoukar Z., Moghimi M., Salehifar E. (2021). A narrative review on adverse effects of dasatinib with a focus on pharmacotherapy of dasatinib-induced pulmonary toxicities. Blood Res..

[52] Cruz-Bermúdez A., Laza-Briviesca R., Vicente-Blanco R.J., García-Grande A., Coronado M.J., Laine-Menéndez S., Palacios-Zambrano S., Moreno-Villa M.R., Ruiz-Valdepeñas A.M., Lendinez C. (2019). Cisplatin resistance involves a metabolic reprogramming through ROS and PGC-1α in NSCLC which can be overcome by OXPHOS inhibition. Free Radic. Biol. Med..

[53] Xu L., Wang W., Zhang X., Ke H., Qin Y., You L., Li W., Lu G., Chan W.-Y., Leung P.C.K. (2019). Palmitic acid causes insulin resistance in granulosa cells via activation of JNK. J. Mol. Endocrinol..

