# Supplementary material for: Small-Molecule Tyrosine Kinase Inhibitors Modulate Glucose Handling in C2C12 Cell Line In Vitro: A Mechanistic Study
Source: Pharmaceuticals (Basel). 2025 Sep 26;18(10):1445. doi: 10.3390/ph18101445 (PMC12566657; doi:10.3390/ph18101445)
Supplement: Supplementary file 1 [file pharmaceuticals-18-01445-s001.zip › pharmaceuticals-3753590-supplementary.pdf]

Actual reading mmol/L			mean	std
21.8	20.5	20.6	20.96667	0.723418
11.5	11.3	11.7	11.5	0.2
6.4	5.9	5.9	6.066667	0.288675
3.2	3.2	3.1	3.166667	0.057735
1.4	1.5	1.5	1.466667	0.057735

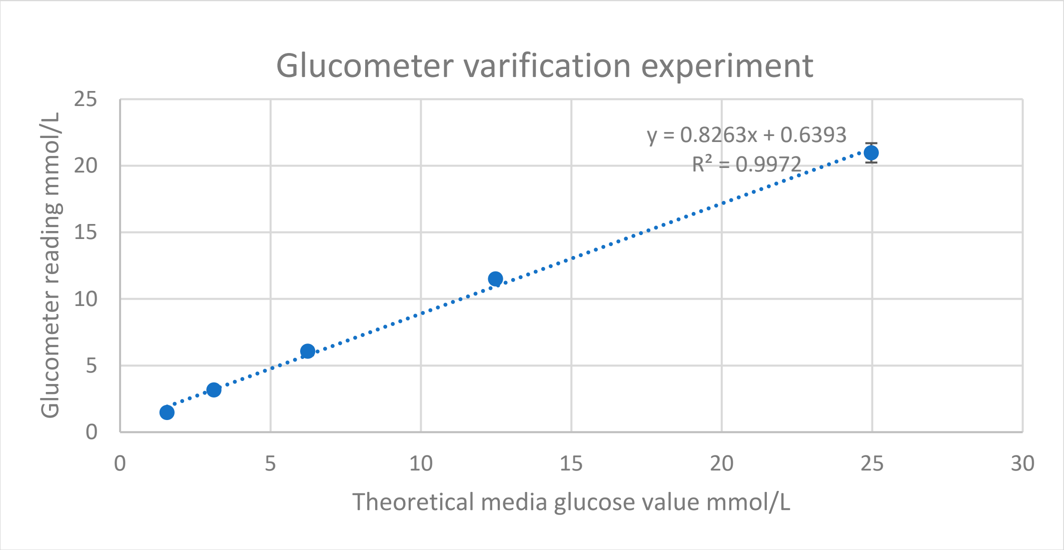

Supplementary Figure S1 shows the glucometer validation for media glucose concentration measurement Experiment; theoretical glucose concentration vs. glucometer glucose reading. The R2 value observed from the plot was 0.9972. The R2 is very close to 1, indicating that the regression line approximates the actual data very well. This means that the glucometer readings are statistically similar to the theoretical glucose concentrations, hence the glucometer was used to estimate cellular glucose uptake. These findings validate the use of glucometers and test strips for relative comparisons of glucose concentrations.
